# Exceptional point enhanced nanoparticle detection in deformed Reuleaux-triangle microcavity

**DOI:** 10.1007/s12200-024-00131-5

**Published:** 2024-08-08

**Authors:** Jinhao Fei, Xiaobei Zhang, Qi Zhang, Yong Yang, Zijie Wang, Chuanlu Deng, Yi Huang, Tingyun Wang

**Affiliations:** Key Laboratory of Specialty Fiber Optics and Optical Access Networks, Joint International Research Laboratory of Specialty Fiber Optics and Advanced Communication, Shanghai Institute for Advanced Communication and Data Science, School of Communication and Information Engineering, Shanghai University, Shanghai, 200444 China

**Keywords:** Exceptional point, Deformed microcavity, Nanoparticle detection, Far-field pattern

## Abstract

In this paper, we propose a deformed Reuleaux-triangle resonator (RTR) to form exceptional point (EP) which results in the detection sensitivity enhancement of nanoparticle. After introducing single nanoparticle to the deformed RTR at EP, frequency splitting obtains an enhancement of more than 6 times compared with non-deformed RTR. In addition, EP induced a result that the far field pattern of chiral mode responses significantly to external perturbation, corresponding to the change in internal chirality. Therefore, single nanoparticle with far distance of more than 4000 nm can be detected by measuring the variation of far field directional emission. Compared to traditional frequency splitting, the far field pattern produced in deformed RTR provides a cost-effective and convenient path to detect single nanoparticle at a long distance, without using tunable laser and external coupler. Our structure indicates great potential in high sensitivity sensor and label-free detector.

**Graphical Abstract:**

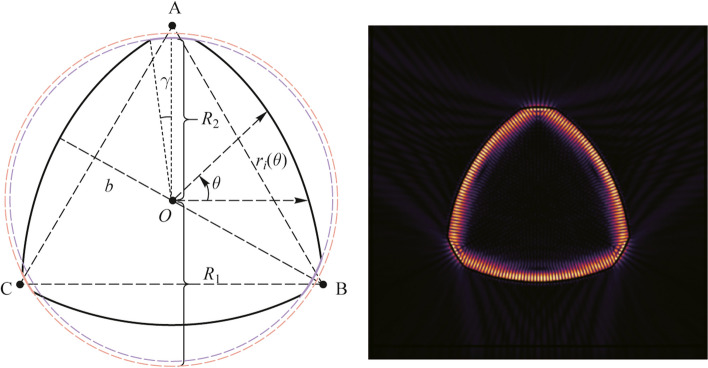

## Introduction

In the past several decades, optical whispering-gallery mode (WGM) microcavities have been widely studied due to small mode volume and high quality (*Q*) factors [1], and a variety of applications have been developed in microcavity sensors [2–4], low threshold lasers [5, 6], cavity quantum electrodynamics [7, 8], nonlinear optics [9–11], etc. At the same time, WGM microcavities provide an ideal platform to explore many phenomena such as non-Hermitian physics [12], exceptional point (EP) [13–15], PT symmetry breaking [16, 17] and resonance-assisted tunneling [18, 19]. In general, EP in the circular microcavity requires two scatters to form [12, 20]. The first scatter is used to break the rotational symmetry of the microcavity and induces mirror symmetry in microcavity. Consequently, the degenerated WGMs split into even and odd modes, and another scatter will coalesce two eigenvalues and corresponding eigenvectors to form EP. However, it is difficult to fine-tune two scatters in a circular microcavity. Due to the degeneracy of deformed microcavity is broken, scatterer number can be reduced to form EP when only the mirror symmetry is held in microcavity [10]. It has been proved that the square root topology characteristic of the frequency splitting near an EP arouses particular interest for the enhancement of nanoparticle detection [21, 22] and realization of optical chirality [17, 23].

The light emission direction is isotropic in the traditional circular microcavity. To realize anisotropic emission and efficiently collect light, the light emission need to be tailored into specific direction. Recently, directional emission has been reported in different microcavity systems, such as microcavity with defects [24], D-shape microcavity [25], limaçon microcavity [26], externally coupled microcavity [22] and microcavity with point scatter [27]. Deformed microcavities are more flexible in morphology and function, but have relatively low *Q* factors and limit the frequency detecting sensitivity of nanoparticle [28]. Thanks to directional emission, the far field pattern (FFP) spectra provide an additional way to detect nanoparticle by internal optical chirality. Hence, chirality plays an important role in directional emission for nanoparticle detecting [29, 30] and nonreciprocal light propagation [31–33]. Up to now, it has been applied to detect nanoparticle with ultra-sensitivity in various deformed microcavities such as spiral microcavity [22, 34], deformed circular square microcavity [21], and optical gyroscopes in limaçon microcavity [35].

In this paper, we propose a deformed Reuleaux-triangle resonator (RTR) with corner-cuts. Phase space structure of the fundamental mode is investigated to obtain strong directional emission and evanescent field at corner-cuts. Due to the square root topology around EP, frequency splitting sensitivity of nanoparticles detection has been tremendously enhanced. According to the close relationship between internal chirality and FFP, the maximum nanoparticle sensing distance and sensitivity of deformed RTR are obtained. Compared to previous studies, our Reuleaux-triangle Microcavity with broken rotation symmetry can form EP with only single scatterer. Thus, the introduction of RTR contributes to simplifying the EP formation and improving the adjustment flexibility.

## Mode performance in Reuleaux-triangle resonator

**Fig. 1:**
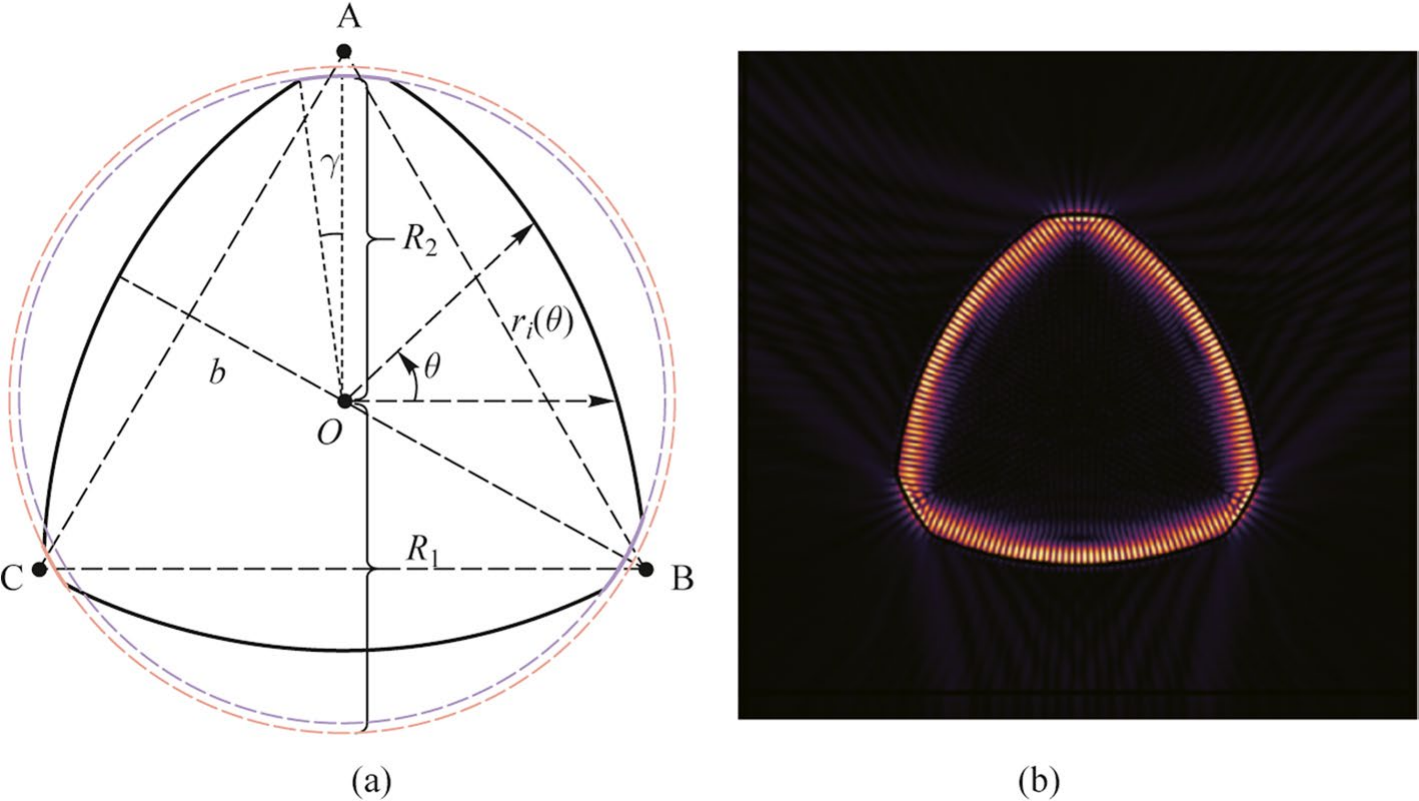
Schematic diagram of a Reuleaux-triangle resonator (RTR) with corner-cut. **a** Plane view of 2D model. **b** Resonant mode in RTR

The Reuleaux triangle is consisted with three rounded arcs similar to a triangle, which is another shape of constant width similar to the circle and has been studied in several applications of robotic leg [36], optical chaotic characteristic [37] and antireflective surface [38]. Besides, the Reuleaux-triangle resonator (RTR) is an ideal platform to study the chaotic and non-Hermitian characteristics of microcavity with unique advantages [37]. In this article, our RTR is used to realize chirality mode at EP for high sensitivity nanoparticle detection. The 2D section of the proposed structure and the corresponding fundamental mode are shown in Fig. 1a and b. The three corners are cut by the purple dashed circle with the radius $R$, and the black solid curve is the actual boundary of RTR with the origin arcs on behalf of corner-cuts. The polar equation of the RTR boundary $\Gamma$ is defined as

1 $$\gamma = \left| {\arctan \left( {\frac{{p^{2} - 2}}{{\sqrt { - p^{4} + 8p^{2} - 4} }}} \right)} \right| - \frac{\uppi }{6},\quad 0 < p \le 1 \, ,$$

2 $$\Gamma=\left\{\begin{array}{l}r_1(\theta)=\sqrt{\frac{2b^2}3+\frac{b^2}4(\text{cos}\theta+\frac{\sqrt3}3\text{sin}\theta)^2}-\frac b2(\text{cos}\theta+\frac{\sqrt3}3\text{sin}\theta),\theta\in\lbrack11\uppi/6+\gamma,2\uppi\rbrack\cup\lbrack0,\uppi/2-\gamma\rbrack,\\r_2(\theta)=\sqrt{\frac{2b^2}3+\frac{b^2}4(\text{cos}\theta-\frac{\sqrt3}3\text{sin}\theta)^2}-\frac b2(\text{cos}\theta-\frac{\sqrt3}3\text{sin}\theta),\theta\;\in\lbrack\uppi/2+\gamma,7\uppi/6-\gamma\rbrack,\\r_3(\theta)=\sqrt{\frac{2b^2}3+\frac{b^2}3(\text{sin}\theta)^2}+\frac{\sqrt3}3b{\text{sin}}\theta,\theta\;\in\lbrack7\uppi/6+\gamma,11\uppi/6-\gamma\rbrack,\\r_4(\theta)=R_{1,2},\theta\in\lbrack m\uppi/6-\gamma,m\uppi/6+\gamma\rbrack,m=3,7,11.\end{array}\right.$$

The parameter $R_{1,2} = pb/\sqrt 3$ decides the size of two different corner-cuts, where $\gamma$ represents the corresponding half arc angle shown in Eq. (1) and Fig. 1b. The functions of ${r}_{i}\left(\theta \right)$ dominated by *b* and $\gamma$ belong to three long circular arcs and corner-cuts in Eq. (2).

To investigate the modes characteristics in RTR, FEM (finite element method) simulation is utilized to analyze TE polarized modes. The effective refractive index of RTR is *n*. Maxwell’s equation can be expressed as a scalar mode equation [39]. The commercial software COMSOL MULTIPHYSICS is chosen to calculate resonance modes, where the eigenfrequency module can obtain the complex frequency $KR$ and the quality factor $Q=-\text{Re}(KR)/\left[2\text{Im}\left(KR\right)\right]$ [14]. The RTR material is EpoCore with refractive index ${n}_{1}=1.575$ and surrounded by air.

**Fig. 2:**
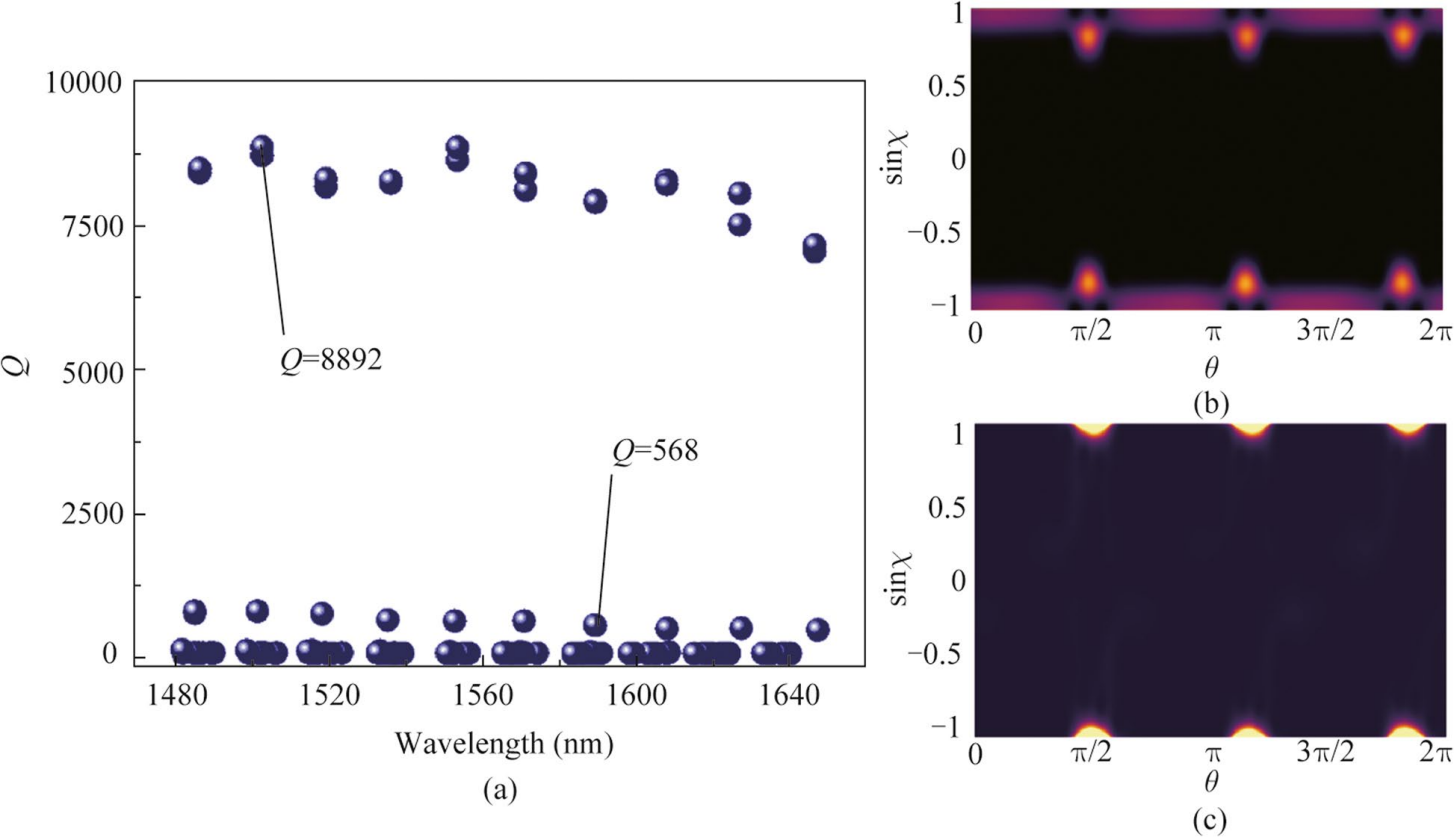
TE polarized modes of the RTR with $b=30\ \upmu\text{m}$ and $p=0.905$. **a** Relationship of *Q* and wavelength. **b** Husimi map ${\mathcal{H}}_{1}^{\text{inc}\left(\text{em}\right)}$ of the high *Q* mode, with $\text{sin}\chi >0$ (CCW) and $\text{sin}\chi <0$ (CW). **c** Husimi map ${\mathcal{H}}_{0}^{\left(\text{em}\right)}$

Figure 2a indicates a complex frequency plane of RTR with fixed parameter of $b=30\ \upmu\text{m}$ and $p=0.905$. It is obvious that a gap exists between long-lived (high *Q* factor of 8086) and short-lived (low *Q* factor around 568) modes, which is advantageous for modes identification. Note that the high *Q* factor modes correspond to the fundamental mode in Fig. 1b, and only one family of long-lived modes can survive in RTR. To demonstrate the mode distribution in phase space, the wave functions can be projected in phase space on the boundary $\Gamma$ via Husimi functions. The Husimi functions along the boundary of RTR ($j=1$, interior boundary; $j=0$, exterior boundary) with the Birkhoff coordinates $\left( {s,\text{sin}\chi } \right)$ can be calculated in Ref. [40]. In the Husimi mapping of resonant mode, the Husimi components of incident wave and reflective wave can be equivalent to ${\mathcal{H}}_{1}^{\text{inc}\left(\text{em}\right)}$. The Husimi map ${\mathcal{H}}_{{1}}^{{\text{inc}\left( {\text{em}} \right)}}$ is shown in Fig. 2b with the complex frequency of $KR=95.535-0.005\text{i}$ with the equivalent intensities in the counterclockwise (CCW) and clockwise (CW) component. $\theta =0$ represents the positive *X*-axis direction of the microcavity. The highest Husimi intensities are located at the corner-cuts, while the second highest intensity is located in period-3 stable island. The Husimi map ${\mathcal{H}}_{{0}}^{{\left( {\text{em}} \right)}}$ in Fig. 2c reveals the emitting intensity along the boundary, which reveals the energy leakage mainly at corner-cuts with strong evanescent field. Meanwhile, light energy is well confined in period-3 islands.

## High chirality for enhancement of sensitivity

As previously stated, deformed microcavities have a natural advantage to build EPs due to the mirror symmetry [21]. The sensitivity of microcavity can be further improved by EPs in parameter space. Around an EP, mode splitting characteristic reveals square-root topology. Thus, for a small perturbation such as nanoparticle, frequency splitting in EPs is expected larger than diabolic point (DP). From this perspective, a simplified formation mechanism of high chirality in deformed boundary of RTR with only one scatterer is proposed. All RTRs are under single-mode operation.

**Fig. 3:**
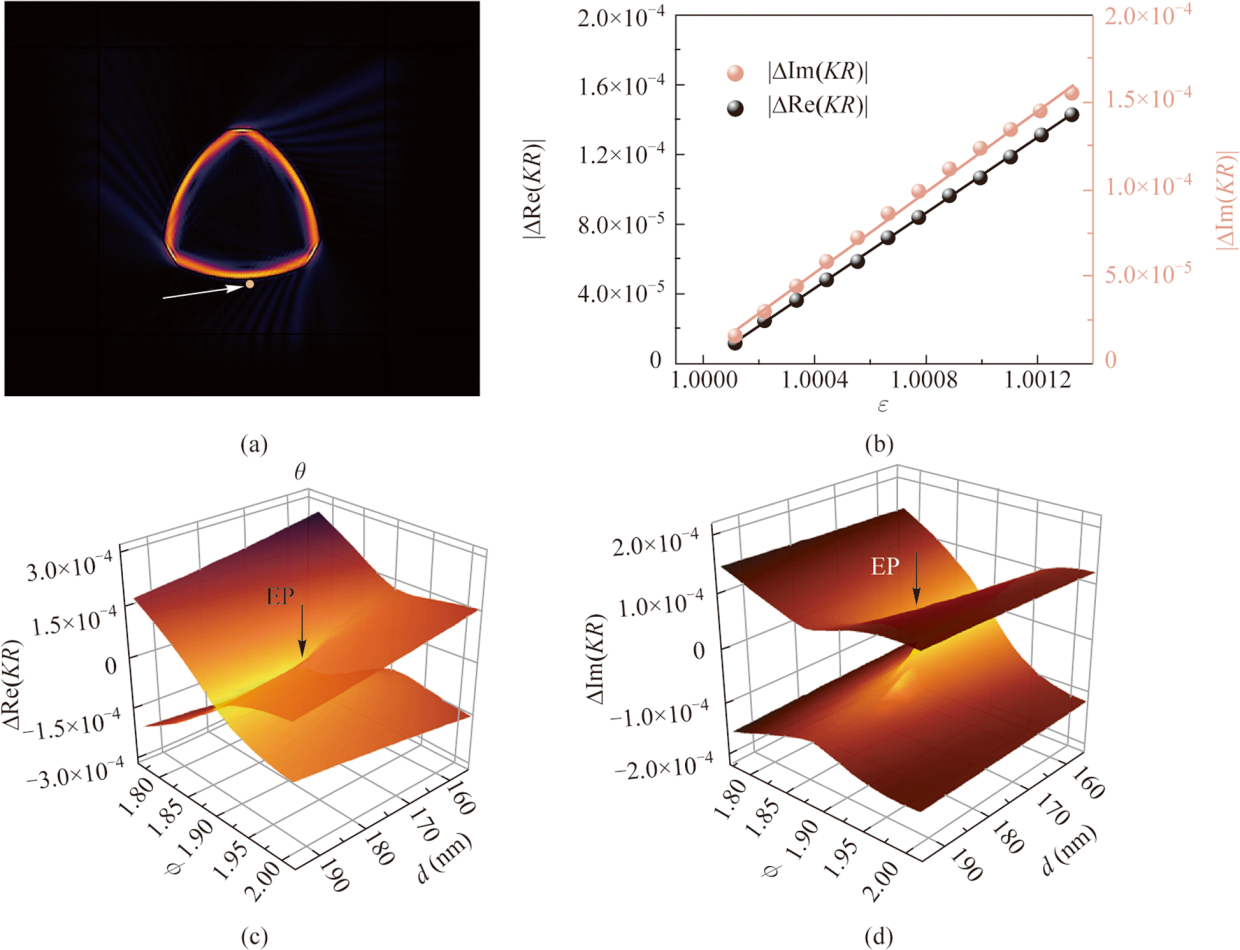
**a** Field distribution of CW mode in deformed RTR near EP. The white arrow shows the scatterer. **b** Real and imaginary parts of frequency splitting $|\Delta KR|$ versus the deformation parameter $\varepsilon$. **c** and **d** Real and imaginary parts of complex frequency of two modes around an EP in ($\Phi , d)$ parameter space. The smallest splitting point near an EP is marked in a black arrow

The rotational symmetry of RTR is broken by cutting the original boundary with the parameter of $$b=30\ \upmu\text{m}$$, using two different circles of radius $${R}_{1}$$ and $${R}_{2}$$ shown in Fig. 1. Due to the *R*_1_ different to *R*_2_, the corner-cuts size of the purple edge is different from the red edge, and the RTR only embed the mirror symmetry. The deformed parameter $$\varepsilon$$ is defined as $${R}_{1}/{R}_{2}$$, and $${R}_{2}$$ is fixed at $$0.905b/\sqrt{3}$$. We define the relationship of $${b/\sqrt{3}>R}_{1}>{R}_{2}$$. Then, the generated WGM modes split into a pair of even and odd parity modes due to the mirror reflection symmetry in the deformed RTR. The frequency splitting $$|\Delta KR|$$ versus $$\varepsilon$$ is shown in Fig. 3b. Both of the real and imaginary parts of $$|\Delta KR|$$ indicate linear splitting with the increase of $$\varepsilon$$ from 1 to 1.0012, owing to a universal phenomenon of finite initial frequency splitting in deformed microcavities. Subsequently, only single scatterer with a refractive index of 1.46 and radius of $$120\ \text{nm}$$ is introduced to couple the pair of even and odd modes into an EP. The scatterer’s position is controlled by a parameter space ($$\Phi , d$$) consisting of the angle away from vertical axis of deformed RTR ($$\Phi )$$ and the distance from boundary of microcavity ($$d)$$. The field distribution of an EP with the disappearing of wave nodes is shown in Fig. 3a. Figure 3c and d show the mean value of complex frequency splitting as the functions of $${{(\Phi }},d)$$ parameter space to confirm the characteristic of square-root topology near an EP [15]. EP position at $$(\Phi ,d){ = }\left( {{1}{\text{.897}}^{{\text{o}}} {,177}{\text{.7}}} \right)$$ is identified by black arrow.

**Fig. 4:**
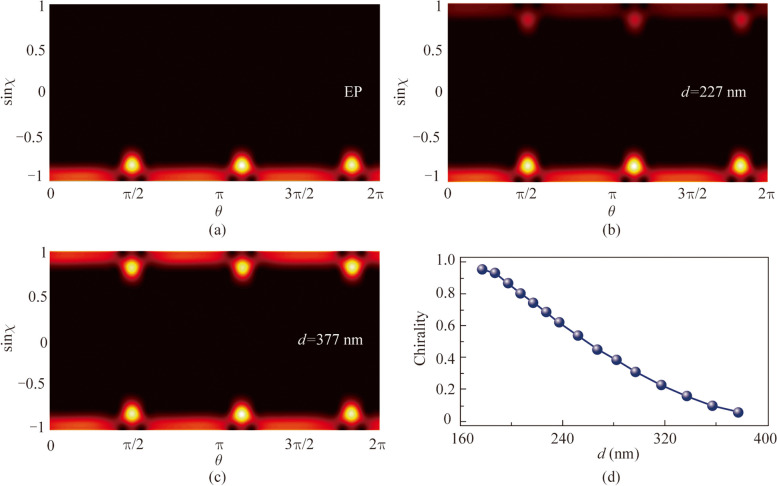
Husimi function (**a**) at EP shown in Fig. 3a, (**b**) at the scatterer distance of 227 nm, and (**c**) at the scatterer distance of 377 nm. **d** Chirality as a function of scatterer distance

Husimi distributions of fundamental mode changed with the scatterer parameter *d* are shown in Fig. 4a−c, respectively. Besides, the Husimi projection can be utilized to characterize the chirality of resonance modes by Eq. (3).

3 $$\alpha =1-\frac{{\sum }_{\text{CCW}}\text{Husimi}}{{\sum }_{\text{CW}}\text{Husimi}}.$$

The ${\sum }_{\text{CCW}}\text{Husimi}$ and ${\sum }_{\text{CW}}\text{Husimi}$ are the total distribution probability of the CCW and CW component, respectively. The charity $\alpha$ calculated as 0.952 is indicative of high chirality mode at EP shown in Fig. 4a. Husimi function is totally dominated by CW component. The RTR slightly deviates from EP point with $\alpha$ reducing to 0.698, and the intensity of CCW component is weaker than CW component when $d=227\ \text{nm}$. While at $d=377\ \text{nm}$, RTR is away from EP and $\alpha$ is close to 0, with the almost equivalent intensities of CCW and CW components. Figure 4d shows the chirality as a function of *d*, where the point of the highest chirality corresponds to the EP.

**Fig. 5:**
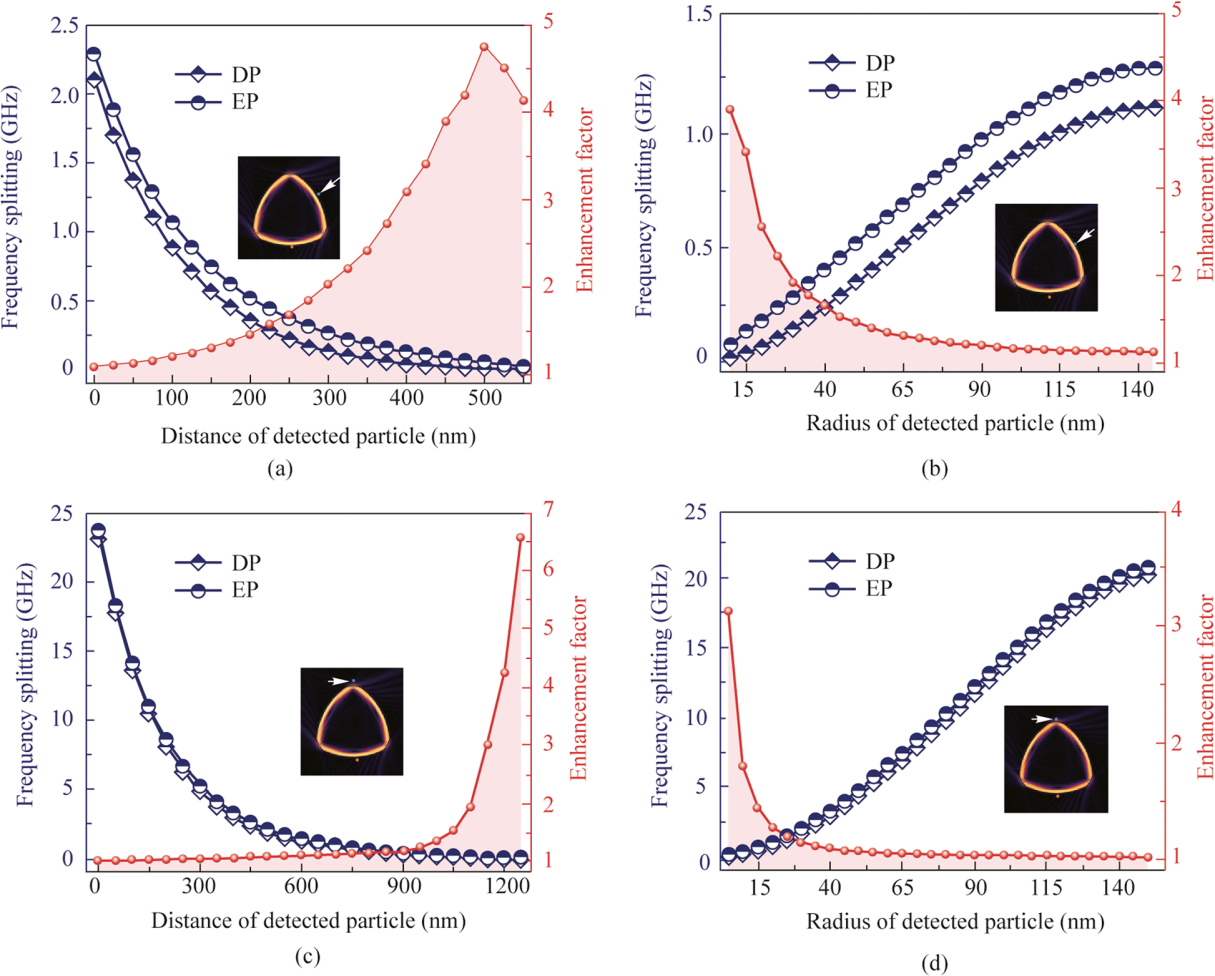
Numerical simulation of nanoparticle detection with $\varepsilon$ equals 1.001. The frequency splitting and enhancement factor with the distance of nanoparticle and different nanoparticle sizes respectively when the particle is located on (**a** and **b**) the long arc edge, (**c** and **d**) the top corner-cut

Thus, the high-sensitivity frequency splitting response with high chirality mode is conducted under different sizes and distances of detection nanoparticles in simulation, both in EP and DP systems. Deformed parameter $\varepsilon$ is fixed to 1.001. The traditional DP sensor has the similar microcavity boundary with the EP sensor but without the single scatterer and boundary deformation, which is shown in Fig. 5a. Mechanically, the proportional frequency splitting for a DP sensor subjected to a perturbation $\varepsilon$ reveals that the larger splitting response in EP system, especially for a weak $\varepsilon$ [20]. The insert in Fig. 5a and b shows the detected nanoparticle located on the right long arc edge with angular position of 30°. Radius of a nanoparticle is fixed at 100 nm. The $\varepsilon$ strength is increased by changing the distance *d* from 550 to 0 nm with the sensing results shown in Fig. 5a. The coupled modes are driven away from an EP after the introducing of detected nanoparticle, which results in the frequency splitting increasing with the decreasing of *d*, both in DP and EP sensors. Nevertheless, the EP sensor’s square root topology characteristic results in a maximum enhancement factor $|\Delta {\omega }_{\text{EP}}/\Delta {\omega }_{\text{DP}}|$ of about 5 at $d=500\ \text{nm}$. The nanoparticle size detection is subsequently changed from 5 to 150 nm with the fixed distance at 100 nm to control the perturbation strength. Similarly, the frequency splitting in EP and DP systems increase with the increased radius of nanoparticle. For the small radius of 5 nm corresponding to weak $\varepsilon$, the enhancement factor achieves 4, consistent with theoretical analysis.

Furthermore, the evanescent field intensity on the three corner-cuts is significantly stronger than the long arc edge one by observing the Husimi map in Fig. 4a. The maximum splitting response of RTR may decrease when the nanoparticles are detected on the long arc edges. Thus, the sensing results are carried out in Fig. 5c and d after selecting the corner-cuts as the detecting arc edges. The configurations of detected nanoparticles are similar to Fig. 5a and b, respectively. As a result, the corner-cuts have shown remarkable improvement in nanoparticle sensing with an enhancement of 23.65 GHz in distance detection and 20.70 GHz in radius detection, which are about 10 times and 16 times better than before, respectively. Moreover, the effective sensing length is improved by 2.5 times to achieve 1250 nm, demonstrating superior advantage of long detecting distance. As a result, we conclude that using corner-cuts as the sensing edges is better for sensing. When the detected particle is far away from the RTR or the particle radius is extremely small, the sensing sensitivity benefits from EP. In other cases, the sensing sensitivity of EP is comparable with DP.

## Far-field detection of nanoparticle

In the discussion of Section 3, EP can improve the detection sensitivity of nanoparticle. However, frequency splitting is difficult to detect in experiment. Due to the fact that internal mode chirality can be observed by external far field, we examine the far-field pattern (FFP) for single nanoparticle detection.

**Fig. 6:**
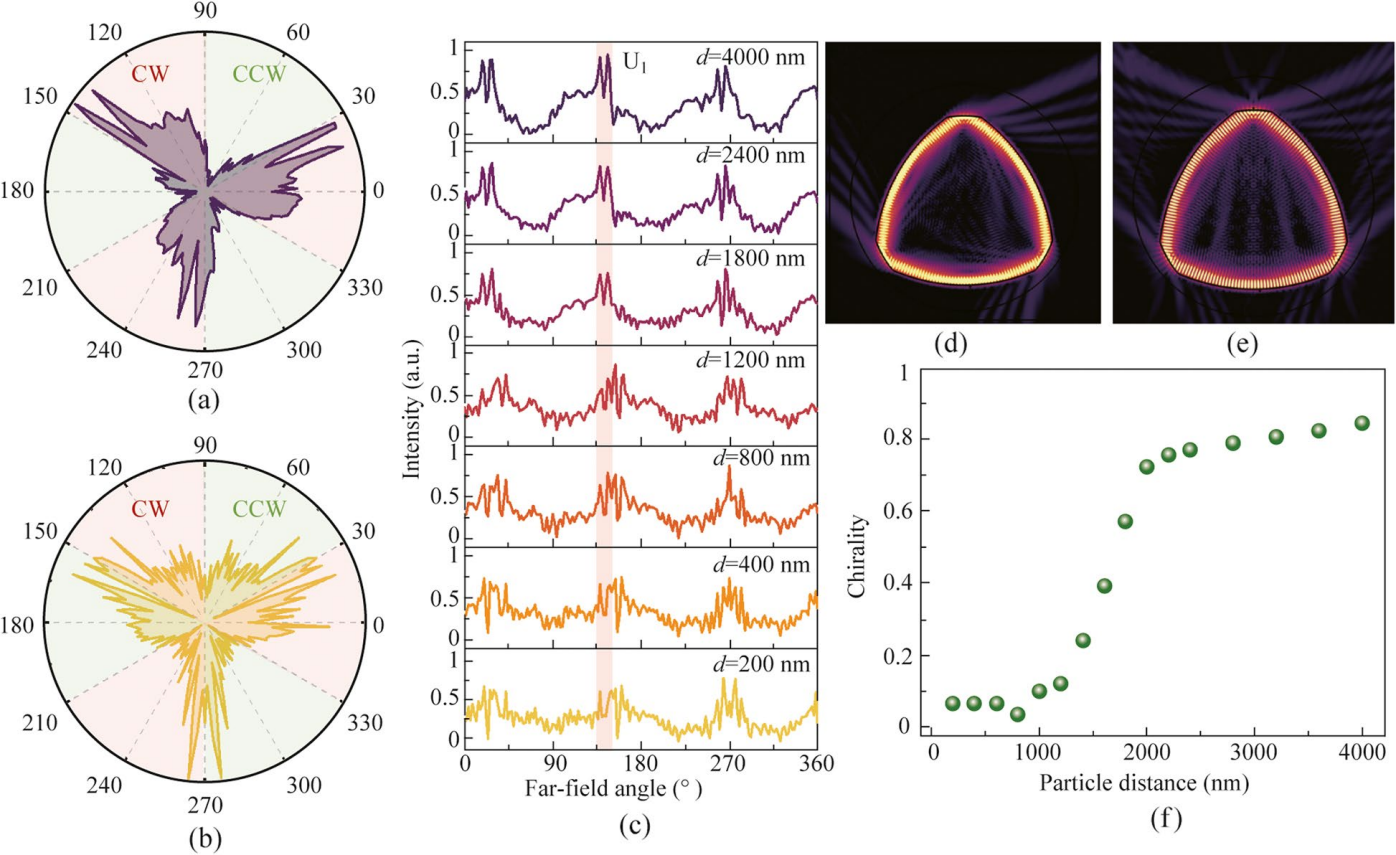
Far-field pattern of based mode with the detection distance *d* of **a** 4000 nm and **b** 200 nm. **c** Far-field patterns with different nanoparticle distances. The lines are vertically shifted for a clear view. **d** and **e** Field distributions correspond to (**a** and **b**). **f** Far-field chirality as a function of particle detection distance

If a detected nanoparticle closes to the RTR, it is effect on resonant mode and chirality may be equivalent to the scatterer in EP. Thus, the mode chirality and FFP will change with the disturbance induced by nanoparticle. Based on this principle, we have studied in simulation to reveal the FFP response with the detecting distance (*d*) of nanoparticle. Nanoparticle is placed above the corner-cuts, and RTR has tuned in EP shown in Fig. 3a. Figure 6a depicts the emission intensity under angular distribution in FFP, where $d=4000$ nm indicates weak disturbance. Due to the rotational symmetry of 120°, we define the red region as CW component and the green region as CCW component. We can find that the directional emission is dominated by CW direction along 20°, 140° and 260° induced by EP, while the emission intensity in CCW direction is obviously weaker. Corresponding mode field is shown in Fig. 6d, with agreement with FFP results. Figure 6b shows the FFP of $d=200$ nm, and the emission intensity reveals the same intensity in CW and CCW directions with the corresponding mode field shown in Fig. 6e. The strong scattering induced by nanoparticle will break EP, results in disappearance of chirality and non-directional emission. Figure 6c shows parts of the far field intensity with different *d*. The emission beam at 140° marked as U_1_ will gradually weak as the decrease of nanoparticles distance. When *d* decreases from 4000, 1200 and 200 nm, the beam intensity is decreased from 96.7%, 73.6% and 57.3%, respectively. Similar pattern applies to another two emission beams at 20° and 260°, and the emission beams gradually appear in the CCW direction. The chirality $\alpha$ as the function of distance *d* is shown in Fig. 6f. The chirality of resonance mode will decrease rapidly with the decrease of nanoparticle distance *d* when *d* is smaller than 2400 nm. When $d=4000$ nm, $\alpha$ is 0.843, which is smaller than chirality 0.952 at EP because of the initial small disturbance induced by nanoparticle. When $d=2400$ nm, $\alpha$ is 0.771. Meanwhile, when $d=1200$ nm, $\alpha$ is 0.12. Chirality will be less than 0.1, when $d<800$ nm.

Therefore, in addition to frequency splitting, the changes of chirality and FFP in RTR provide a convenient way to detect a single nanoparticle. The frequency splitting is relatively weak when the detected nanoparticle is far away from RTR, although EP has already enhanced the maximum frequency response sensitivity. Due to the *Q* factor decrease of deformed RTR, linewidth of resonance mode is extended. Thus, weak frequency splitting is difficult to measure in experiments, which limits its practical application. Thanks to the FFP characteristic of independent on *Q* factor, we can use the change of chirality to easily detect nanoparticle instead of frequency splitting. Compared to previous researches on frequency splitting for nanoparticle detection, our research depends on the angular distribution of FFP and does not require high spectrum resolution. Thus, using far field micro-laser provides an economical and robust scheme for nanoparticle detection.

## Conclusions

In summary, we have proposed a deformed Reuleaux-triangle resonator (RTR) with corner-cuts to simplify the EP forming process and to detect a single nanoparticle through the far field pattern. Fundamental mode in RTR indicates directional emission and strong evanescent field at corner-cuts, by using the phase space analysis. Deformed RTR embedded mirror reflection symmetry can form EP with single scatterer, and realize high chirality mode. After introducing a nanoparticle at EP, the deformed RTR performs superior sensitivity enhancement up to 6.5 times than the non-deformed RTR at DP. Meanwhile, we have confirmed the fact that the far field pattern of chirality mode responds drastically to the disturbance of external nanoparticle. Therefore, the RTR can effectively detect a single nanoparticle at distance up to 4000 nm, with the highest sensitivity around 1800 nm. Consequently, our nanoparticle detector indicates great potential in applications of high sensitivity sensing and long-distance bio/chemical sensor.

## Data Availability

Data underlying the results presented in this paper are not publicly available at this time but may be obtained from the authors upon reasonable request.
